# Microbial regulation of the L cell transcriptome

**DOI:** 10.1038/s41598-017-18079-2

**Published:** 2018-01-19

**Authors:** Tulika Arora, Rozita Akrami, Ramona Pais, Linda Bergqvist, Bengt R. Johansson, Thue W. Schwartz, Frank Reimann, Fiona M. Gribble, Fredrik Bäckhed

**Affiliations:** 1 Wallenberg Laboratory and Sahlgrenska Center for Cardiovascular and Metabolic Research, Department of Molecular and Clinical Medicine, Institute of Medicine, University of Gothenburg, Gothenburg, Sweden; 2 Novo Nordisk Foundation Center for Basic Metabolic Research, Section for Metabolic Receptology and Enteroendocrinology, Faculty of Health Sciences, University of Copenhagen, Copenhagen, Denmark; 3 Metabolic Research Laboratories, Institute of Metabolic Science, Addenbrooke’s Hospital, Hills Road, Cambridge, UK; 4 Dept of Rheumatology and Inflammation Research, University of Gothenburg, Gothenburg, Sweden; 5 Institute of Biomedicine, Department of Medical Chemistry and Cell Biology, The Sahlgrenska Academy at Gothenburg University, Gothenburg, Sweden

## Abstract

L cells are an important class of enteroendocrine cells secreting hormones such as glucagon like peptide-1 and peptide YY that have several metabolic and physiological effects. The gut is home to trillions of bacteria affecting host physiology, but there has been limited understanding about how the microbiota affects gene expression in L cells. Thus, we rederived the reporter mouse strain, GLU-Venus expressing yellow fluorescent protein under the control of the proglucagon gene, as germ-free (GF). L_pos_ cells from ileum and colon of GF and conventionally raised (CONV-R) GLU-Venus mice were isolated and subjected to transcriptomic profiling. We observed that the microbiota exerted major effects on ileal L cells. Gene Ontology enrichment analysis revealed that microbiota suppressed biological processes related to vesicle localization and synaptic vesicle cycling in L_pos_ cells from ileum. This finding was corroborated by electron microscopy of L_pos_ cells showing reduced numbers of vesicles as well as by demonstrating decreased intracellular GLP-1 content in primary cultures from ileum of CONV-R compared with GF GLU-Venus mice. By analysing L_pos_ cells following colonization of GF mice we observed that the greatest transcriptional regulation was evident within 1 day of colonization. Thus, the microbiota has a rapid and pronounced effect on the L cell transcriptome, predominantly in the ileum.

## Introduction

The gut microbiota is considered an environmental factor that regulates host metabolism by interacting with different tissues, both locally and systemically, *via* microbiota-derived signals and metabolites^1,2^. The primary interface of host-microbiota interactions is the intestinal epithelium^3^. Cells of the intestinal epithelium consist of three functional groups: proliferating stem cells, absorptive enterocytes and secretory cells including enteroendocrine, goblet and Paneth cells^4^. Enteroendocrine cells comprise 1% of the intestinal epithelium but constitute the largest network of endocrine cells in the body expressing a wide variety of hormones^5^. Among the enteroendocrine cells, L cells are of significant interest as they secrete glucagon like peptide-1 (GLP-1) and peptide YY (PYY), hormones with multiple paracrine and endocrine effects^6^, and therapeutic potential in the treatment of type 2 diabetes^7^. In addition, L cells are found along the longitudinal axis of the intestine and are sensitive to luminal nutritional stimuli^8^ and microbiota-derived products such as short chain fatty acids (SCFAs)^9^ and secondary bile acids^10^.

To date, several studies have addressed how the microbiota interacts with dietary fibers and that the resulting SCFAs induce colonic proglucagon expression and plasma GLP-1 levels^11,12^. Furthermore, comparing germ-free (GF) and conventionally raised (CONV-R) mice revealed that GF mice, unexpectedly, had increased expression of colonic proglucagon resulting in increased circulating GLP-1 levels^13,14^. The increased levels of GLP-1 appeared to have primarily a paracrine function suppressing the intestinal transit rate to allow more time for energy harvesting in the absence of microbes and fermentation on a fiber-rich diet^13^. The diffuse localization of L cells has so far restricted investigations to tissue level expression or use of *ex vivo* methods, and thus posed difficulties in understanding their biology at the cellular level. Recent development of transgenic GLU-Venus mice driving expression of yellow fluorescent protein (YFP) under the proglucagon promoter has facilitated a greater understanding of intestinal L cells at the cellular level^15^. So far, GLU-Venus mice have been characterized in CONV-R mice under standard chow^15^ and high fat diet conditions^16^. Here, we derived GLU-Venus mice under GF conditions and investigated 1) how the gut microbiota regulates the transcriptome of ileal and colonic L cells and 2) what transcriptional responses are induced in the L cells of ileum and colon during course of colonization of GF GLU-Venus mice.

## Results

### The gut microbiota regulates gene expression profiles of L cells in a site-specific manner

**Figure 1:**
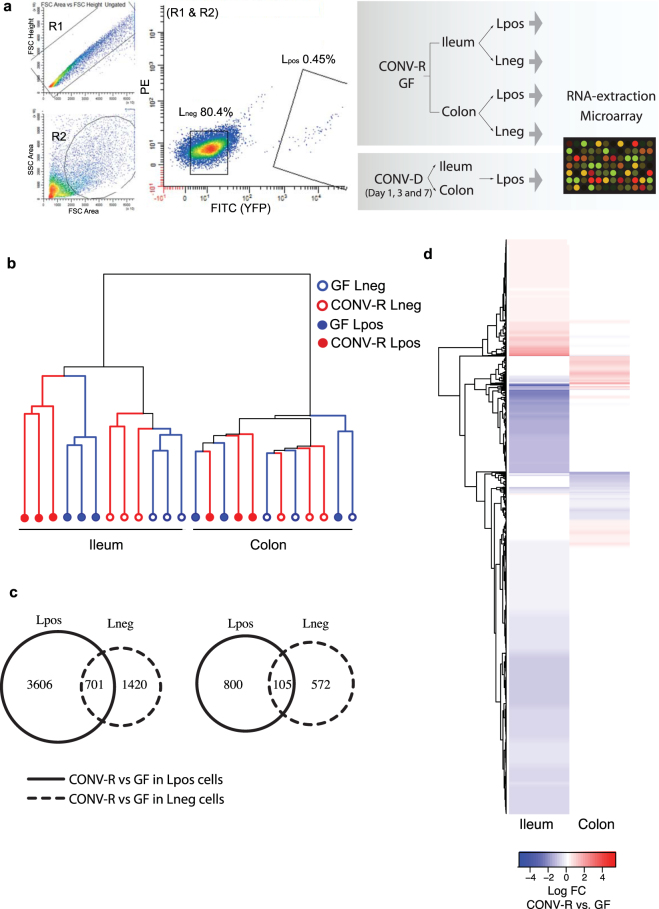
Microbiota-responsive genes in L_pos_ cells from ileum and colon. (**a**) Representative flow cytometry plot showing gate settings; R1 and R2 gates were first applied to exclude doublets and dead cells, respectively. The YFP-positive L cell (L_pos_) and YFP-negative heterogeneous populations (L_neg_) were then sorted from the ileum and colon of transgenic GLU-Venus mice under conventionally raised (CONV-R) and germ-free (GF) conditions. L_pos_ cells were also obtained from mice conventionalized for 1, 3 and 7 days (CONV-D). Sorted cell populations from all groups were subjected to RNA extraction and microarray analysis. (**b**) Hierarchical clustering dendrogram of whole-transcriptome expression profiles obtained using DNA microarrays. (**c**) Venn diagram showing the number of microbiota-dependent genes (p_adj_ < 0.05 for CONV-R vs GF) in L_neg_ (dotted black), L_pos_ populations (solid black) in ileum (left) and colon (right) of GLU-Venus mice. (**d**) Heat map showing log fold change in expression of significantly altered genes in CONV-R versus GF comparison in colonic and ileal L_pos_ cells.

To investigate the effect of the gut microbiota on the gene expression profile of L cells, we rederived GLU-Venus mice as GF and used flow cytometry followed by microarray to analyze the transcriptome of proglucagon (*Gcg*)-expressing YFP-positive (L_pos_) cells and heterogeneous YFP-negative (L_neg_) cells from the ileum and colon of CONV-R and GF GLU-Venus mice (Fig. 1a). Hierarchical clustering revealed a clear separation of samples first by tissue (ileum versus colon), then by cell type (L_pos_ versus L_neg_), and finally according to the bacterial status (GF versus CONV-R); clustering by bacterial status was more evident in the ileum than the colon (Fig. 1b).

First, we focused on the expression of known L cell hormones in L_pos_ and L_neg_ cells from the ileum and colon of GF and CONV-R GLU-Venus mice. As expected, the expression of hormones was higher in L_pos_ cells compared with L_neg_ cells (Supplementary Fig. S1a). While the expression of genes such as, proglucagon (*Gcg)*, peptide YY (*Pyy)*, cholecystokinin (*Cck)*, secretin (*Sct)* and neurotensin (*Nts)* was high in L_pos_ cells from both the ileum and the colon, gastric inhibitory peptide (*Gip)* and insulin-like peptide 5 (*Insl5)* were only expressed at high levels in L_pos_ cells from the ileum and colon, respectively (Supplementary Fig. S1a); however, expression of these hormones did not differ between GF and CONV-R GLU-Venus mice. Of note, *Gcg*, *Pyy*, *Cck*, *Sct*, *Nts and Gip* in L_pos_ cells from the ileum and *Gcg*, *Pyy*, *Cck*, *Sct*, *Nts and Insl5* in L_pos_ cells from the colon were among the most abundant of all the genes analyzed (Supplementary Fig. S1b), which likely resulted in saturation of the assay and thus microbial regulation could not be observed. In contrast, microbial regulation was observed only for the relatively low expressing gene encoding pancreatic polypeptide (*Ppy*) in the colon: its expression was higher in L_pos_ cells from CONV-R compared with GF GLU-Venus mice **(**Supplementary Fig. S1a).

Next, we used a linear regression test to score for differential expression depending on cell type and colonization to identify genes that were regulated by the microbiota in L_pos_ cells from the ileum and colon. After excluding microbially regulated genes that were common to the L_neg_ and L_pos_ populations, we identified 3606 (log fold change: −5.4 to +3.0) and 800 (log fold change: −2.6 to +2.1) genes that were regulated by the microbiota (p_adj_ < 0.05) in L_pos_ cells from the ileum and colon, respectively (Fig. 1c). In ileal L_pos_ cells, of the genes that were differentially regulated (n = 3606), 76%were downregulated in CONV-R compared with GF GLU-Venus mice (Fig. 1d). We also observed that expression of the genes encoding olfactory, vomeronasal and taste receptors was higher in ileal L_pos_ cells from CONV-R compared with GF GLU-Venus mice (Table S1). In contrast, expression of *Gpbar1* (G-protein coupled bile acid receptor1, also called *Tgr5*) was lower in L_pos_ cells from CONV-R compared with GF GLU-Venus mice (Table S1). In colonic L_pos_ cells, of the genes that were differentially regulated (n = 800), 45% were downregulated in CONV-R compared with GF GLU-Venus mice (Fig. 1d).

### The gut microbiota regulates the functional capacity of L cells in a site-specific manner

**Figure 2:**
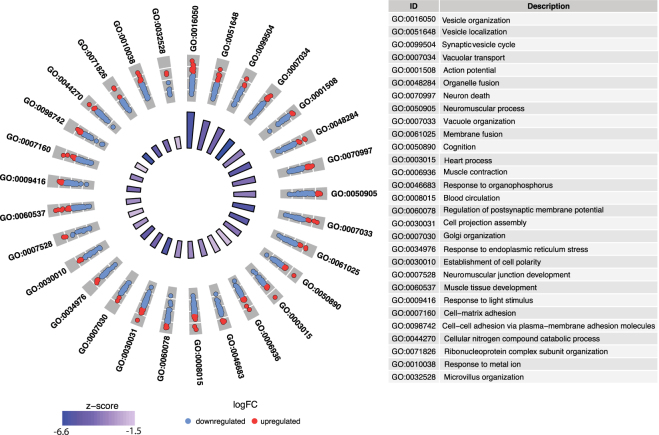
Microbiota-responsive gene functions in ileal L_pos_ cells. Gene ontology (GO) enrichment analysis of microbiota-regulated genes in CONV-R versus GF comparison in L_pos_ population from ileum. The outer circle shows scatterplot for log fold change of the assigned up- (red) or downregulated (blue) genes within each GO category. The bars in inner circle indicate gradient of z-scores (calculated by the number of upregulated genes minus the number of downregulated genes divided by the square root of the count). The length of each bar indicates the extent of significance (adjusted p-value) of the downregulated GO category.

To assess the potential functional impact of the microbiota on the transcriptome in L cells from the ileum and colon, we performed gene ontology (GO) enrichment analysis on the genes significantly regulated by the microbiota in L_pos_ cells from the ileum and colon. In the ileum, 29 GO categories were significantly downregulated in L_pos_ cells from CONV-R compared with GF GLU-Venus mice (Fig. 2). The most significantly downregulated GO categories were vesicle organization and vesicle localization (Fig. 2). In addition, the GO categories synaptic vesicle cycle, action potential and Golgi organization were downregulated in L_pos_ cells from CONV-R compared with GF GLU-Venus mice (Fig. 2). The key marker genes related to vesicle organization and synaptic vesicle cycle were downregulated in L_pos_ cells from CONV-R compared with GF GLU-Venus mice were those encoding synaptophysin (*Syp*), synaptotagmins, Rab and SNAP proteins (Table S1). These results suggest that biological functions related to intracellular vesicle localization are affected by colonization status. Expression of the GO category cellular nitrogen compound catabolic process was also lower in L_pos_ cells from CONV-R versus GF GLU-Venus mice (Fig. 2), suggesting higher utilization of nitrogenous compounds in GF GLU-Venus mice. In L_pos_ cells from the colon, we observed that there was no significant regulation of GO categories (after FDR correction) by the gut microbiota. However, there was a trend towards  enrichment of GO functions related to response to metal ions and nutrient levels, and organic cyclic compound catabolic process in L_pos_ cells from CONV-R versus GF GLU-Venus mice (Table S2).

### Ileal L cells have higher intracellular GLP-1 content

**Figure 3:**
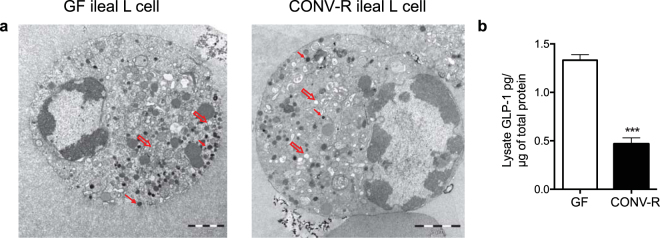
The gut microbiota regulates intracellular vesicles and GLP-1 content in ileal L_pos_ cells. (**a**) Electron microscope images of ileal L_pos_ cells from GF and CONV-R GLU-Venus mice (n = 2–3). Red arrows indicate the densely packed vesicles and open arrows indicate the open (empty) type vesicles, scale 2 μm. (**b**) Intracellular GLP-1 content (normalized to total protein) in the lysate from primary crypt cultures of ileum of GF and CONV-R GLU-Venus mice (n = 3–4). Data are mean ± SEM. ***p < 0.001 indicates significance in CONV-R versus GF comparison.

Since we observed significant microbial downregulation of genes related to vesicle localization in ileal L cells, we investigated whether this observation was paralleled by functional effects. By performing transmission electron microscopy, we observed decreased numbers of densely packed vesicles in CONV-R compared with GF ileal L_pos_ cells (Fig. 3a). In addition, basal intracellular GLP-1 content was significantly reduced in ileal primary crypt cultures from CONV-R compared with GF GLU-Venus mice (Fig. 3b), consistent with the decreased numbers of secretory vesicles.

### Kinetics of transcription regulation of L cells by the gut microbiota

**Figure 4:**
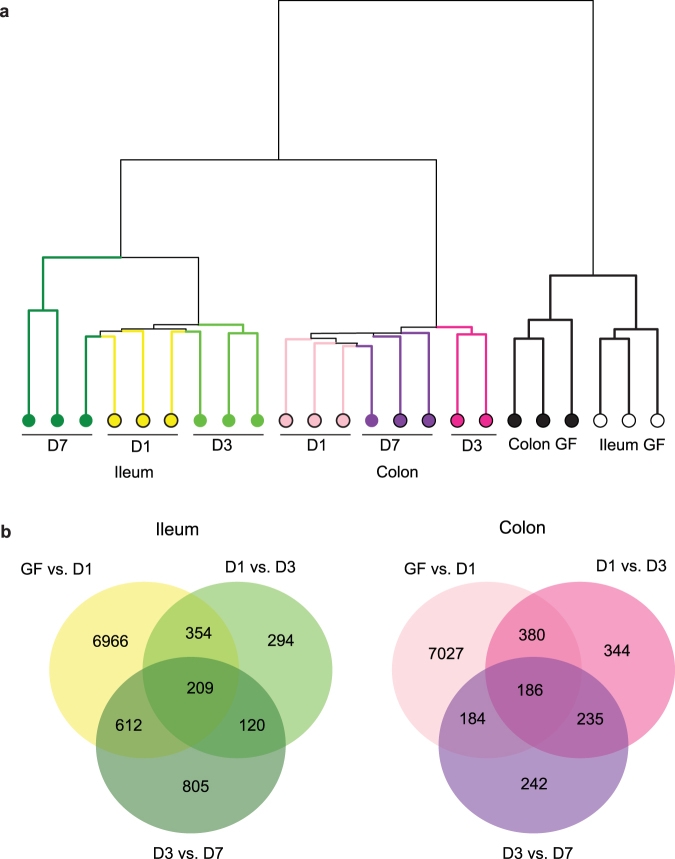
Ileal and colonic L cells respond fast to colonization by unfractionated microbiota. (**a**) Hierarchical clustering dendrogram of whole-transcriptome expression profiles obtained using DNA microarrays. (**b**) Venn diagram showing the number of significantly altered genes in D1 versus GF, D3 versus D1 and D7 versus D3 comparisons in L_pos_ populations from ileum and colon.

To elucidate the temporal sequence of the transcriptional responses elicited by the gut microbiota in L cells, GF mice were colonized with unfractionated microbiota from a CONV-R mouse and the L_pos_ populations were sorted from colon and ileum using flow cytometry at day 1, 3 and 7 after colonization. Hierarchical clustering revealed that GF tissue samples clustered separately from colonized and that samples from colonized mice were first separated by tissue and then by the day of colonization status (Fig. 4a). By performing pairwise comparisons of the gene profiles in L_pos_ cells obtained before and at different time points after colonization, we observed that the major changes in gene expression occurred at day 1 after colonization in both the ileum and colon (Fig. 4b). Furthermore, we observed that more genes were regulated between day 3 and day 7 after colonization in L_pos_ cells from the ileum than from the colon (Fig. 4b).

Next, we focused on the genes that were exclusively regulated by the microbiota in L_pos_ cells from the ileum (n = 3606) and colon (n = 800) that were identified when comparing CONV-R and GF GLU-Venus mice (Fig. 1c) and monitored their regulation over the colonization period. We observed that 1469 of the 3606 genes shown to be regulated in ileal L_pos_ cells from CONV-R versus GF mice were also regulated in ileal L_pos_ cells after 1 day of colonization (p_adj_ < 0.05), and most of these genes were downregulated (Supplementary Fig. S2). GO enrichment analysis revealed that GO categories belonging to vesicle organization and localization, synaptic vesicle cycle and Golgi organization were significantly downregulated in ileal L_pos_ cells from mice after 1 day of colonization (Table 1). Moreover, the GO category synaptic vesicle cycle was also downregulated in ileal L_pos_ cells from D7 versus D3 colonized mice (Table 1).

**Table 1:** Gene ontology (GO) enrichment analysis of microbiota regulated genes in D1colonized versus GF and D7 versus D3 colonized comparisons in L_pos_ population from ileum.

Day 1 versus GF				
ID	Description	Adj. p value	Gene Count	z-score
GO:0016050	Vesicle organization	2.42e-03	35	−0.65
GO:007030	Golgi organization	6.43e-03	18	−0.47
GO:0030010	Establishment of cell polarity	8.68e-03	19	−0.65
GO:0061025	Membrane fusion	9.52e-03	27	−0.62
GO:0099504	Synaptic vesicle cycle	1.53e-02	17	−0.50
GO:0048284	Organelle fusion	2.36e-02	24	−0.67
GO:0051648	Vesicle localization	2.52e-02	24	−0.39
**Day 7 versus Day 3**				
GO:0099504	Synaptic vesicle cycle	5.81e-03	10	−0.80
GO:0032615	Interleukin-12 production	3.75e-02	6	−0.67

In addition, we observed that 328 of the 800 genes shown to be regulated in colonic L_pos_ cells from CONV-R versus GF mice were also regulated in colonic L_pos_ cells after 1 day of colonization (p_adj_ < 0.05), and 71% of these genes were downregulated (Supplementary Fig. S2). GO enrichment analysis of these genes revealed no significant regulation of GO categories in colonic L_pos_ cells from mice after 1 day of colonization.

## Discussion

The gut microbiota regulates many aspects of intestinal homeostasis including vascularization^17^, permeability^18^ and activation of innate and adaptive immunity^19^ that translates into diseased states such as inflammatory bowel disease and metabolic impairments in the host^4^. Microbial regulation in intestine has focused on intestinal epithelial cells but not in the specialized cells of intestine, such as enteroendocrine cells. Here, we showed specific regulation of L cells in the ileum and proximal colon by the gut microbiota with more genes being regulated by the microbiota in L_pos_ cells from the ileum than from the colon. We also observed microbially induced downregulation of biological functions associated with vesicle organization and synaptic vesicle cycle specifically in L_pos_ cells isolated from the ileum of GLU-Venus mice. The gene expression profiles at the transcriptional level were confirmed as electron microscopy revealed a reduced number of densely packed vesicles in L_pos_ cells from the ileum of CONV-R compared with GF GLU-Venus mice. In addition, intracellular GLP-1 content was lower in primary crypt cultures from CONV-R compared with GF GLU-Venus mice. Furthermore, we showed that the gene expression profiles in ileal L cells responded fast to the microbial colonization; one-day colonization of GF GLU-Venus mice resulted in expression profiles similar to CONV-R GLU-Venus mice.

L cells are highly responsive to nutrients and microbiota-derived metabolites, which stimulate hormone secretion^6^. In contrast to our previous study where we found that the microbiota predominantly affected proglucagon expression in the colon^13^, we here observed that the microbiota had its major effect on L cells in the ileum. This finding is consistent with previous studies demonstrating that the gut microbiota has a more profound effect on transcriptional responses in the ileum than in the colon^20,21^, potentially due to direct contact of ileal enterocytes and enteroendocrine cells with mucosal microbiota that extends into the lumen in the ileum, whereas colonic cells are separated from luminal bacteria by a mucus layer that is devoid of bacteria^22^. Interestingly, we observed that the microbiota predominantly suppressed genes in the ileum. In contrast, genes encoding small molecule receptors had increased expression in CONV-R mice, suggesting that these cells may be more responsive to microbial metabolites and induce signaling. We observed relatively small effects by the microbiota on the transcriptome on colonic L-cells, which is in line with that microbiota predominantly regulates energy availability in the colon, e.g. butyrate, and that the colonic L-cells mainly mediate this signal in a paracrine fashion^13^.

We also observed that the gut microbiota downregulated biological functions related to vesicle organization and localization specifically in the ileal L cells. We corroborated this finding by demonstrating reduced numbers of densely packed vesicles in L_pos_ cells and decreased intracellular GLP-1 content in ileal primary culture from CONV-R compared with GF GLU-Venus mice. GF mice are known to have higher levels of GLP-1 in portal blood than CONV-R mice^13^. Since both the small intestine and proximal large intestinal regions drains into the portal vein, higher expression of genes related to vesicle organization in L_pos_ cells may suggest that ileum also contributes towards the difference in portal levels of GLP-1 between GF and CONV-R mice.

We also observed that biological functions related to synaptic vesicle cycle and action potential were also downregulated in L_pos_ cells from the ileum of CONV-R compared with GF GLU-Venus mice. This may indicate that L_pos_ cells are in direct connection with neurons, in agreement with previous studies^23^. However, it may also merely reflect altered vesicle formation and hormone release in L cells from CONV-R mice.

We observed that L_pos_ cells from both the ileum and colon exhibited altered transcriptional activity as early as one day post microbiota colonization of GF GLU-Venus mice. The rapid increase in SCFA following one day colonization of GF mice^13^ may contribute to altered transcriptional profiles in L_pos_ cells, demonstrating the rapidity in responses mediated by the microbiota and presumably microbiota derived metabolites. Taken together, our findings suggest that the microbiota produces rapid and tissue- specific regulation of the L cell transcriptome and that there is a need of generating small and large intestine specific knockouts to delineate the respective role for L cells in these tissues.

## Material and Methods

### Mice

Transgenic GLU-Venus mice previously used to characterize the transcriptional capacity of L cells^15^, were rederived as GF as described in^24^. The GF mice were maintained in flexible plastic film isolators under 12 h dark light cycles and were fed autoclaved normal chow and water *ad libitum*^24^. Male mice aged 8–14 weeks under CONV-R and GF conditions were used. For colonization experiments, cecal contents from a CONV-R GLU-Venus mouse was homogenized in PBS buffer supplemented with reducing solution (0.02 M Na_2_S and 1% cystein dissolved in NaHCO_3_ buffer) and orally gavaged to GF GLU-Venus mice after a 4 h fast. Transplanted mice were maintained in autoclaved individual ventilated cages with sterile bedding and fed autoclaved food and water *ad libitum*for 1, 3 and 5 days respectively. All procedures in mice were approved by the Ethics Committee on Animal Care and Use in Gothenburg, Sweden.

### Preparation of single cell suspension

GF and CONV-R GLU-Venus mice were killed by cervical dislocation and the distal 10 cm of the small intestine or first half of colon was opened, fecal material removed and washed in 6 changes of PBS in a 6-well plate. To prepare single cell suspensions from ileum, tissue was digested with 0.26 Wünsch units Liberase (Roche) in DMEM (with high glucose) at 37 °C for 20 min and shaken vigorously every 5 min to dissociate cells. The digestion was repeated 4 times with fresh Liberase, cells were pooled, passed through a 100 µm pore diameter cell strainer, pelleted at 1200 rpm for 5 min and resuspended in DMEM (with high glucose) supplemented with 10% fetal bovine serum.

To prepare single cell suspensions from colon, tissue was incubated in 25 ml of EDTA/DTT solution (3 mM EDTA/0.05 mM DTT solution in PBS) at 37 °C for 30 min. The EDTA/DTT solution was removed, tissue was shaken vigorously with fresh 10 ml PBS three times to dissociate crypts and centrifuged at 500 rpm for 5 min. The cell pellet was digested in 20 ml of 0.2% (w/v) Pancreatin (Sigma) in PBS. Following incubation, the cell suspension was diluted with an equal volume of PBS, centrifuged at 1500 rpm for 10 min and resuspended in DMEM (with high glucose) supplemented with 10% fetal bovine serum.

### Flow cytometry assisted sorting of L cells

A SY3200 sorter (Sony Biotechnology) was used to separate YFP expressing L_pos_ from non-YFP expressing L_neg_ cells in both colonic and ileal single cell suspensions. Settings: 70uM nozzle, 50–52 psi, 77.3 kHz, plates charged with 4000 V. Single cells were selected by their forward scatter area and forward scatter height. L_pos_ cells were selected by their relative fluorescence at 500–550 nm (excited by a 488 nm laser). The cells were gated to exclude doublets and dead cells, and were sorted against YFP vs. PE channels to collect only YFP expressing L_pos_ populations directly into RNALater (Sigma) at room temperature. A heterogenous non-YFP expressing L_neg_ population was also collected for each sample. The gating strategy was changed minimally between samples (Fig. 1).

### RNA extraction

The cell pellets were homogenized in RLT buffer supplemented with 2-mercaptoethanol using QiaShredder (Qiagen, Hilden, Germany). RNA was extracted using the RNeasy micro Kit with on-column DNase I treatment (Qiagen). RNA concentration and quality were evaluated using capillary electrophoresis on a 2100 Bioanalyzer with RNA 6000 Pico kit (Agilent Technologies, Santa Clara, CA, USA).

### Microarray

Total RNA (500 pg) from each sample was used to generate amplified and biotinylated sense transcript cDNA from the entire expressed transcriptome according to the Nugen Technologies (San Carlos, CA, USA) Ovation® Pico WTA System V2 (M01224v2) and Encore Biotine Module (M01111v5). GeneChip ST Arrays (GeneChip Mouse Gene 2.0 ST Array) were hybridized for 16 h in a 45 °C incubator, rotated at 60 rpm. The arrays were then washed and stained using the Fluidics Station 450 and finally scanned using the GeneChip Scanner 3000 7 G according to the GeneChip Expression Wash, Stain and Scan Manual (PN 702731 rev. 3, Affymetrix, Santa Clara, CA, USA).

### *Ex vivo* primary culture

The distal 10 cm of the small intestine was dissected out and washed with PBS. The muscle layer was removed under dissection microscope. Tissue was digested with 0.4 mg/ml Collagenase XI, centrifuged at 300xg, and resuspended in Dulbecco’s modified Eagle’s medium (25 mM glucose) supplemented with 10% FBS, 2 mM L-glutamine, penicillin, and streptomycin. Aliquots were plated on matrigel-coated 24-well plates and incubated for 24 hours days at 37 °C, 5% CO_2_. Cultures were incubated in saline buffer containing 0.1% fatty acid-free BSA for 2 hr at 37 °C. Cells were then treated with lysis buffer containing: 50 mM Tris-HCl, 150 mM NaCl, 1% IGEPAL-CA 630, 0.5% deoxycholic acid, and one tablet of complete EDTA-free protease inhibitor cocktail (Roche) to extract intracellular peptides. GLP-1 was assayed in cell extract using total GLP-1 Mesoscale discovery kit. Total protein content in cell extracts was measured using the BCA assay.

### Electron microscopy of ileal L cells

Sorted L_pos_ cells were fixed overnight in 1.25% glutaraldehyde + 1% formaldehyde + 0.02% Na azide in 0.1 M Na cacodylate buffer. Fixed cells were rinsed twice in PBS with gentle pelletting at 150 g for 20 min. The final loose pellet was resuspended at 37 °C in 10% (w/v) gelatin in PBS. Aliquots of 40 µl were transferred to minitubes pre-loaded to half their volume with Fluorinert 70 and centrifuged at 1500 rpm for 10 min in a cooling centrifuge. During this time, cells become enriched against the surface of the Fluorinert before the gelatin solidified by cooling. The gelatin cylinders with cells were further stabilized at 0 °C for 1 h and treated as tissue blocks i.e. fixed in the aldehyde mixture and post-fixed in 1% (w/v) osmium tetroxide. After contrasting *en bloc* in 0.5% uranyl acetate, the blocks were dehydrated, infiltrated with Agar 100 epoxy resin and cured by heat. Ultrathin sections were obtained in a Leica UC6 ultramicrotome and were examined in a LEO 912AB electron microscope.

### Data Analysis

The whole-transcript level of the mice genome was measured by MoGene 2.0 ST chips (Affymetrix, Santa Clara, CA, USA). The probe set summarization and normalization was done with Affymetrix expression console software. All the downstream analysis was done in R version 3.1.3 software environment^25^. The probe sets were annotated to ENSEMBL gene reference using MoGene 2.0 ST probe set mapping provided by Affymetrix mogene20 annotation data R-package^26^. The dissimilarities between samples were calculated using Canberra distance metric and the hierarchical clustering was performed using Ward’s hierarchical agglomerative clustering method^27,28^. The differential expression of the genes was assessed by the robust method of Limma (Linear models for microarray and RNA-seq data) R-package^29^. Gene Ontology grouping and enrichment analysis was performed by ClusterProfiler R-package^30^ and using biological processes database from genome wide annotation for mouse R-package (org.Mm.eg.db)^31^. Biological processes from GO annotations were selected at level 5. All the p-values were corrected using Benjamini & Hochberg method^32^. Venn diagrams were plotted using VennDiagram R-package and heatmaps were plotted using ggplot2 R-package^33,34^. GO circle was plotted using GOplot R-package^35^.

### Data availability statement

The datasets generated during this study are available in the ArrayExpress database at EMBL-EBI (www.ebi.ac.uk/arrayexpress) under accession numbers E-MTAB-6322 and E-MTAB-6324. 

## Electronic supplementary material

Supplementary information
