# Supplementary material for: Microbial regulation of the L cell transcriptome
Source: Sci Rep. 2018 Jan 19;8:1207. doi: 10.1038/s41598-017-18079-2 (PMC5775357; doi:10.1038/s41598-017-18079-2)
Supplement: Supplementary file 1 — Supplementary information [file 41598_2017_18079_MOESM1_ESM.pdf]

Supplementary figures and legends

Microbial regulation of the L cell transcriptome

Tulika Arora[1, 2], Rozita Akrami[1], Ramona Pais[3], Linda Bergqvist[4], Bengt R Johansson[5], Thue W Schwartz[2], Frank Reimann[3], Fiona M Gribble[3], Fredrik Bäckhed*[1, 2]

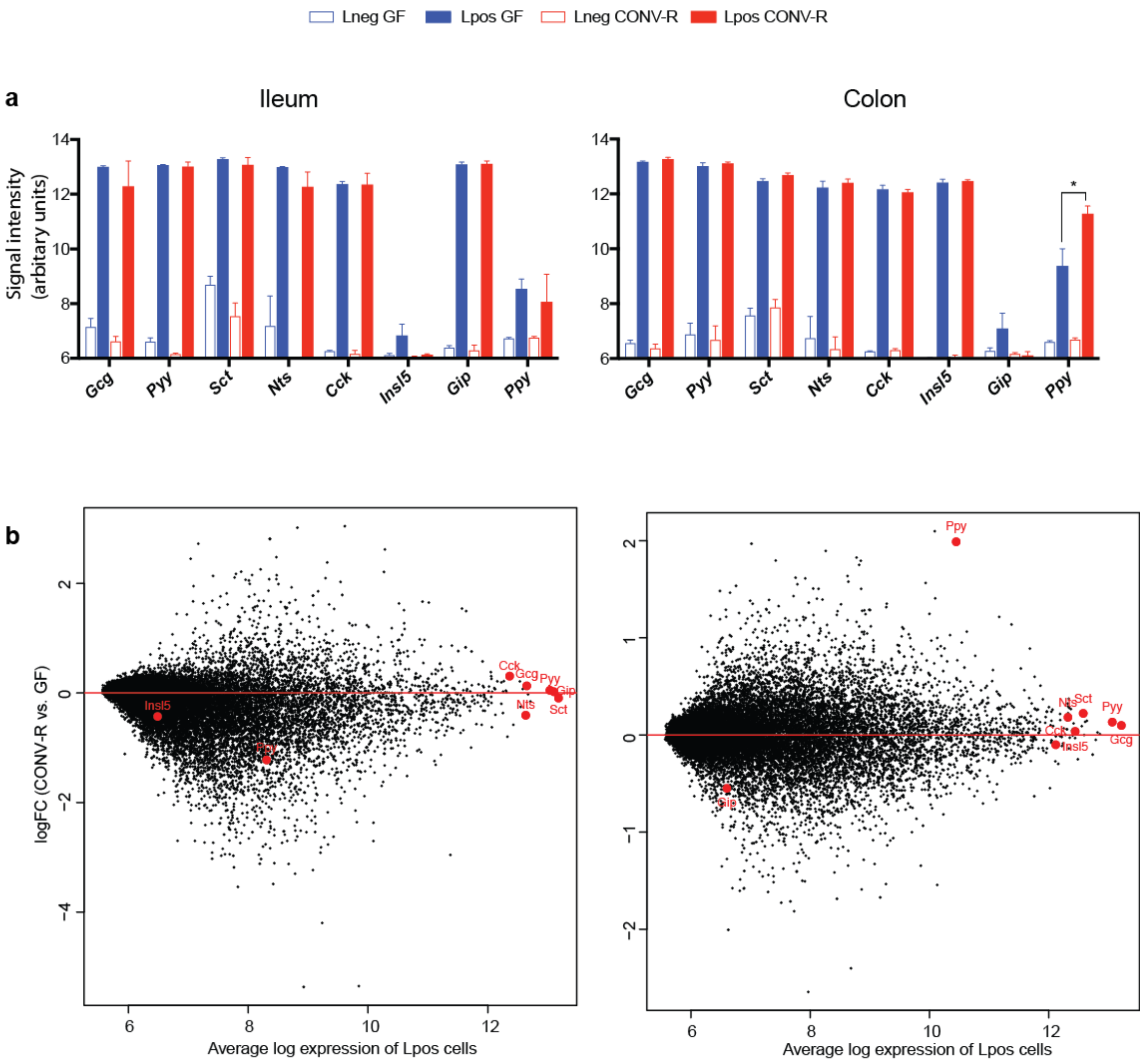

Supplementary figure S1. (a) The expression of genes (expressed as signal intensity) encoding hormones (Gcg,Pyy, Nts, Cck, Insl5, Gip and Ppy) in Lneg and Lpos populations from ileum and colon in GF and CONV-R GLU-Venus mice. *p<0.05 indicates CONV-R versus GF comparison. (b) MA plots showing average log expression of genes against log fold change (log FC) in CONV-R versus GF comparison in Lpos cells from ileum and colon. Each dot respresents a gene and the genes encoding L cell hormones are labelled in red.

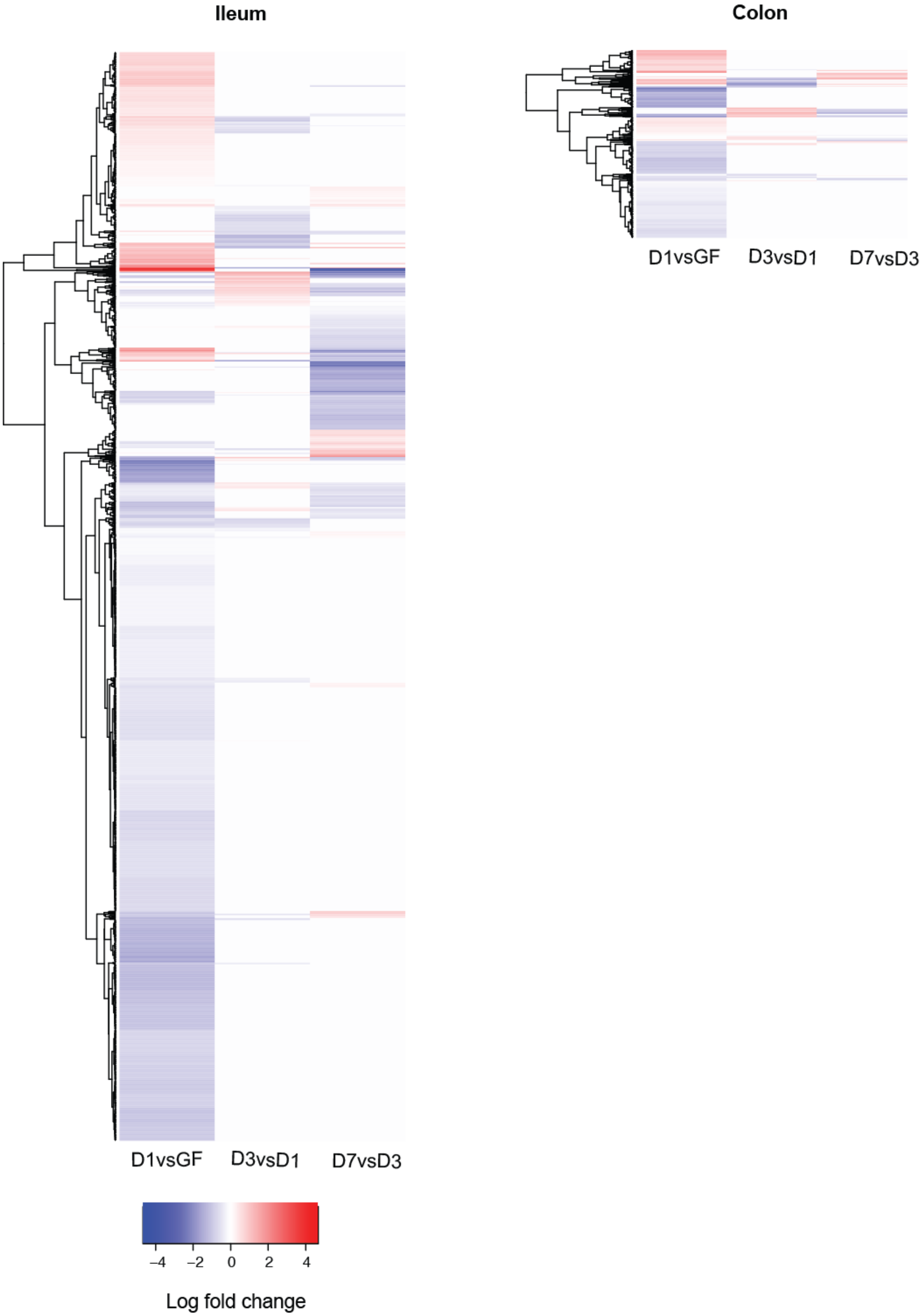

Ileum
Colon
D1vsGF
D3vsD1
D7vsD3
D1vsGF
D3vsD1
D7vsD3
−4
−2
0
2
4
Log fold change

Supplementary figure S2. Expression of microbiota-dependent genes in Lpos populations from ileum and colon (obtained in Fig 2) was monitored over D1, 3 and 7 colonization period. Heat map indicates log fold change in expression of significantly altered genes ($p<0.05$) genes in D1 versus GF, D3 versus D1 and D7 versus D3 comparisons in Lpos populations from ileum and colon.

Genes encoding different receptor groups				
**ID**	**Gene Symbol**	**Description**	**logFC**	**adj.P.Val**
17214162	Gpbar1	G protein-coupled bile acid receptor 1	-3.2336	0.0000
17362138	Gpr137	G protein-coupled receptor 137	-1.3305	0.0003
17541465	Gpr119	G-protein coupled receptor 119	-0.8849	0.0002
17337770	Gpr116	G protein-coupled receptor 116	-0.7207	0.0003
17534982	Gpr112	G protein-coupled receptor 112	-0.5121	0.0017
17357630	Gpr44	G protein-coupled receptor 44	-0.4916	0.0022
17284247	Gpr132	G protein-coupled receptor 132	-0.2734	0.0171
17369340	Gpr107	G protein-coupled receptor 107	-0.1879	0.0126
17535353	Gpr50	G-protein-coupled receptor 50	0.1459	0.0136
17331212	Gpr15	G protein-coupled receptor 15	0.1817	0.0163
17538960	Gpr143	G protein-coupled receptor 143	0.2061	0.0381
17533446	Gpr34	G protein-coupled receptor 34	0.2144	0.0461
17294485	Gpr150	G protein-coupled receptor 150	0.2751	0.0085
17541919	Gpr101	G protein-coupled receptor 101	0.2856	0.0048
17504207	Gpr97	G protein-coupled receptor 97	0.3627	0.0027
17280571	Gpr22	G protein-coupled receptor 22	0.8624	0.0000
17248911	Olfr56	olfactory receptor 56	-0.3573	0.0011
17370376	Olfr368	olfactory receptor 368	-0.2214	0.0118
17387578	Olfr988	olfactory receptor 988	-0.2057	0.0432
17357940	Olfr1495	olfactory receptor 1495	-0.1587	0.0168
17514916	Olfr835	olfactory receptor 835	-0.1520	0.0249
17457981	Olfr448	olfactory receptor 448	-0.1425	0.0222
17299512	Olfr748	olfactory receptor 748	0.1011	0.0267
17249258	Olfr54	olfactory receptor 54	0.1157	0.0372
17494208	Olfr69	olfactory receptor 69	0.1289	0.0282
17246392	Olfr767	olfactory receptor 767	0.1306	0.0262
17372912	Olfr1188	olfactory receptor 1188	0.1323	0.0431
17331230	Olfr178	olfactory receptor 178	0.1331	0.0304
17337625	Olfr133	olfactory receptor 133	0.1424	0.0364
17363062	Olfr1426	olfactory receptor 1426	0.1427	0.0338
17246416	Olfr777	olfactory receptor 777	0.1427	0.0155
17493121	Olfr309	olfactory receptor 309	0.1452	0.0392
17219762	Olfr414	olfactory receptor 414	0.1470	0.0480
17481328	Olfr659	olfactory receptor 659	0.1519	0.0362
17372771	Olfr1022	olfactory receptor 1022	0.1528	0.0365
17514920	Olfr837	olfactory receptor 837	0.1541	0.0148
17252749	Olfr59	olfactory receptor 59	0.1550	0.0443
17494203	Olfr630	olfactory receptor 630	0.1585	0.0378
17372931	Olfr1212	olfactory receptor 1212	0.1595	0.0035
17481153	Olfr564	olfactory receptor 564	0.1605	0.0416

17246422	Olfr801	olfactory receptor 801	0.1614	0.0153
17525720	Olfr945	olfactory receptor 945	0.1623	0.0393
17285641	Olfr1367	olfactory receptor 1367	0.1647	0.0358
17525799	Olfr985	olfactory receptor 985	0.1655	0.0191
17387902	Olfr1223	olfactory receptor 1223	0.1689	0.0257
17374184	Olfr1284	olfactory receptor 1284	0.1705	0.0132
17372906	Olfr1184	olfactory receptor 1184	0.1755	0.0390
17387636	Olfr1040	olfactory receptor 1040	0.1801	0.0481
17357887	Olfr1444	olfactory receptor 1444	0.1881	0.0222
17372927	Olfr1204	olfactory receptor 1204	0.1890	0.0298
17494815	Olfr517	olfactory receptor 517	0.1986	0.0059
17494688	Olfr695	olfactory receptor 695	0.1988	0.0135
17337604	Olfr124	olfactory receptor 124	0.1993	0.0117
17372759	Olfr1014	olfactory receptor 1014	0.1994	0.0049
17481510	Olfr716	olfactory receptor 716	0.2015	0.0282
17387720	Olfr1098	olfactory receptor 1098	0.2019	0.0306
17534667	Olfr1320	olfactory receptor 1320	0.2036	0.0226
17462133	Olfr212	olfactory receptor 212	0.2053	0.0035
17494254	Olfr66	olfactory receptor 66	0.2065	0.0373
17494467	Olfr684	olfactory receptor 684	0.2100	0.0012
17265802	Olfr398	olfactory receptor 398	0.2108	0.0304
17481250	Olfr633	olfactory receptor 633	0.2138	0.0225
17290906	Olfr1368	olfactory receptor 1368	0.2172	0.0076
17486096	Olfr1349	olfactory receptor 1349	0.2208	0.0084
17466416	Olfr458	olfactory receptor 458	0.2208	0.0014
17337608	Olfr125	olfactory receptor 125	0.2256	0.0090
17238642	Olfr763	olfactory receptor 763	0.2264	0.0115
17263185	Olfr323	olfactory receptor 323	0.2397	0.0410
17494443	Olfr666	olfactory receptor 666	0.2427	0.0079
17265761	Olfr1	olfactory receptor 1	0.2525	0.0017
17516120	Olfr145	olfactory receptor 145	0.2621	0.0006
17336071	Olfr55	olfactory receptor 55	0.2637	0.0023
17457977	Olfr449	olfactory receptor 449	0.3191	0.0248
17481141	Olfr557	olfactory receptor 557	0.3348	0.0000
17341326	Vmn2r109	vomeronasal 2, receptor 109	-0.1597	0.0120
17246473	Vmn2r84	vomeronasal 2, receptor 84	0.1042	0.0309
17467181	Vmn1r21	vomeronasal 1 receptor 21	0.1288	0.0204
17459248	Vmn1r37	vomeronasal 1 receptor 37	0.1305	0.0316
17458928	Vmn1r5	vomeronasal 1 receptor 5	0.1321	0.0482
17493053	Vmn2r67	vomeronasal 2, receptor 67	0.1322	0.0144
17234873	Vmn2r81	vomeronasal 2, receptor 81	0.1369	0.0398
17450904	Vmn2r14	vomeronasal 2, receptor 14	0.1388	0.0339
17460749	Vmn1r41	vomeronasal 1 receptor 41	0.1408	0.0466

17333769	Vmn2r95	vomeronasal 2, receptor 95	0.1456	0.0245
17488090	Vmn1r185	vomeronasal 1 receptor 185	0.1461	0.0497
17291064	Vmn1r189	vomeronasal 1 receptor 189	0.1484	0.0347
17486527	Vmn1r84	vomeronasal 1 receptor 84	0.1499	0.0154
17346591	Vmn2r120	vomeronasal 2, receptor 120	0.1507	0.0251
17486055	Vmn1r60	vomeronasal 1 receptor 60	0.1608	0.0133
17462816	Vmn2r23	vomeronasal 2, receptor 23	0.1616	0.0412
17479952	Vmn2r77	vomeronasal 2, receptor 77	0.1753	0.0092
17467187	Vmn1r22	vomeronasal 1 receptor 22	0.1762	0.0057
17486314	Vmn2r42	vomeronasal 2, receptor 42	0.1842	0.0396
17458936	Vmn1r8	vomeronasal 1 receptor 8	0.1885	0.0403
17490031	Vmn2r59	vomeronasal 2, receptor 59	0.1902	0.0006
17479967	Vmn2r79	vomeronasal 2, receptor 79	0.2045	0.0404
17474863	Vmn1r174	vomeronasal 1 receptor 174	0.2249	0.0082
17487619	Vmn1r149	vomeronasal 1 receptor 149	0.2372	0.0041
17434167	Vmn2r122	vomeronasal 2, receptor, 122	0.3261	0.0017
17232172	Taar1	trace amine-associated receptor 1	0.1301	0.0416
17232195	Taar7f	trace amine-associated receptor 7F	0.1524	0.0405
17232178	Taar3	trace amine-associated receptor 3	0.1884	0.0240
**Genes groups involved in synaptic vesicle cycle**				
**ID**	**Gene Symbol**	**Description**	**logFC**	**adj.P.Val**
17532879	Syp	synaptophysin	-0.7412	0.0047
17357486	Syt7	synaptotagmin VII	-1.9621	0.0000
17533234	Sytl5	synaptotagmin-like 5	-1.5187	0.0000
17246163	Esyt1	extended synaptotagmin-like protein 1	-1.1727	0.0011
17279584	Esyt2	extended synaptotagmin-like protein 2	-1.1419	0.0013
17373530	Syt13	synaptotagmin XIII	-0.6979	0.0121
17231174	Syt14	synaptotagmin XIV	-0.3572	0.0428
17340532	Sytl3	synaptotagmin-like 3	0.1687	0.0112
17217536	Syt2	synaptotagmin II	0.2947	0.0323
17485815	Syt5	synaptotagmin V	0.9105	0.0000
17376822	Snap25	synaptosomal-associated protein 25	-1.6392	0.0000
17375083	Snap23	synaptosomal-associated protein 23	-1.3474	0.0032
17263401	Snap47	synaptosomal-associated protein, 47	-0.9653	0.0130
17529417	Snap91	synaptosomal-associated protein 91	-0.7167	0.0000
17279737	Rab10	RAB10, member RAS oncogene family	-1.4537	0.0003
17468705	Rab7	RAB7, member RAS oncogene family	-1.3400	0.0005
17306835	Rabggta	Rab geranylgeranyl transferase, a subunit	-1.0574	0.0000
17515340	Rab3d	RAB3D, member RAS oncogene family	-1.0374	0.0003
17342496	Rab40c	Rab40c, member RAS oncogene family	-1.0242	0.0183
17348238	Rab18	RAB18, member RAS oncogene family	-0.9455	0.0210
17416764	Rab3b	RAB3B, member RAS oncogene family	-0.9226	0.0000

17524827	Rab3d	RAB3D, member RAS oncogene family	-0.9129	0.0000
17397462	Rab33b	RAB33B, member of RAS oncogene family	-0.8971	0.0319
17547998	Rab11b	RAB11B, member RAS oncogene family	-0.8626	0.0008
17228751	Rabgap1l	RAB GTPase activating protein 1-like	-0.8615	0.0020
17342559	Rab11fip3	RAB11 family interacting protein 3 (class II)	-0.8528	0.0004
17282074	Rab15	RAB15, member RAS oncogene family	-0.8482	0.0040
17411635	Rab2a	RAB2A, member RAS oncogene family	-0.7770	0.0115
17480205	Rab30	RAB30, member RAS oncogene family	-0.7277	0.0000
17247533	Rab1	RAB1, member RAS oncogene family	-0.6711	0.0183
17306274	Rab2b	RAB2B, member RAS oncogene family	-0.6137	0.0031
17346840	Rab12	RAB12, member RAS oncogene family	-0.5779	0.0226
17225460	Rab17	RAB17, member RAS oncogene family	-0.5039	0.0011
17380251	Rab22a	RAB22A, member RAS oncogene family	-0.4714	0.0181
17530231	Rab6b	RAB6B, member RAS oncogene family	-0.3752	0.0380
17535984	Rab39b	RAB39B, member RAS oncogene family	-0.3162	0.0019
17527166	Rab39	RAB39, member RAS oncogene family	-0.2207	0.0184
17366055	Rab11fip2	RAB11 family interacting protein 2 (class I)	-0.2054	0.0168
17520922	Rab6b	RAB6B, member RAS oncogene family	-0.1781	0.0250
17544779	Rab9b	RAB9B, member RAS oncogene family	0.3470	0.0020
17502039	Rab3a	RAB3A, member RAS oncogene family	0.9580	0.0075

Supplementary table S1. List of gene groups ($p<0.05$) encoding receptors, and involved in synaptic vesicle cycling in Lpos cells from ileum. LogFC indicate log fold change of gene in GF versus CONV-R comparison

ID	Description	p-value	Adj. p-value	z-score
GO:0008015	Blood circulation	0.00866	0.30081	0.00000
GO:0030031	Cell projection assembly	0.01125	0.31714	0.20851
GO:0042060	Wound healing	0.02925	0.4412	0.47141
GO:1901361	Organic cyclic compound catabolic process	0.01915	0.37754	0.72761
GO:0006936	Muscle contraction	0.0058	0.28599	0.72761
GO:0016050	Vesicle organization	0.01136	0.31714	0.50000
GO:0031667	Response to nutrient levels	0.03076	0.44794	2.32380
GO:0010038	Response to metal ion	0.0088	0.30081	0.53452
GO:0003015	Heart process	0.01079	0.31323	-0.27735
GO:0098780	Response to mitochondrial depolarisation	0.00131	0.18782	2.30940
GO:0006839	Mitochondrial transport	0.04692	0.49775	-1.15470
GO:0009743	Response to carbohydrate	0.04494	0.49775	-0.90453
GO:0051648	Vesicle localization	0.03999	0.48394	1.50756

Supplementary table S2. Gene ontology (GO) enrichment analysis of microbiota-regulated genes in CONV-R versus GF comparison in Lpos population from colon.
